# Contours of the transatlantic regulatory landscape: A research agenda towards the reform of US state pharmacy board disciplinary functions

**DOI:** 10.1016/j.rcsop.2026.100818

**Published:** 2026-06-24

**Authors:** Denzil Johnson, Nkiruka Umaru, Cathal T. Gallagher

**Affiliations:** School of Health, Medicine and Life Sciences, University of Hertfordshire, Hatfield, UK

**Keywords:** Bounded accountability, Comparative law, Pharmacy regulation, Professional discipline, Public protection, Regulatory capture

## 1 Introduction

The crisis in American medical regulation has been well documented both in academic literature and the media,^1^, ^2^, 3., 4., 5, 6, 7, 8. and, while comparable research into other professions is scant, the consequences of inadequate professional discipline within pharmacy are potentially significant. Without effective governmental regulation that prevents unethical or incompetent pharmacists from practising, the risk to patients and the public is obvious.

Regulatory bodies may sometimes act infrequently, slowly, or leniently when addressing professional misconduct in pharmacy. One explanation lies in the structure of professional regulation, particularly in relation to decision-making within disciplinary processes. In American jurisdictions, disciplinary cases are considered by panels that include practising pharmacists who may have substantial professional workloads and limited regulatory training. As a result, they may rely heavily on professional judgment and may be inclined to prioritise remediation and rehabilitation of pharmacists rather than imposing restrictive sanctions. While supporting professional improvement is important, this approach can contribute to disciplinary outcomes that may not always sufficiently prioritise patient safety and public protection.

Pharmacy is regulated through legislative frameworks that define the scope of practice, set standards for entry into the profession through education, training, and registration requirements, and establish professional and ethical standards for safe and effective practice. These legislative frameworks also establish regulatory bodies responsible for overseeing the profession of pharmacy. These regulators develop detailed professional standards, assess applications for registration, investigate complaints, and determine whether pharmacists have breached professional or legal standards. Where violations occur, regulators have the authority to impose sanctions, including conditions on practice, suspension, or removal from the professional register. In this way, pharmacy regulators are responsible for both establishing professional rules and applying them when assessing individual cases of professional misconduct or impaired fitness to practise.

To highlight some of the potential issues arising from the current model of disciplining pharmacist in the US, we will use the United Kingdom of Great Britain and Northern Ireland (UK) as an exemplar. The UK was chosen because regulation of pharmacists in the UK has recently been reformed by the creation of the General Pharmaceutical Council (GPhC) and the modernisation of the Pharmaceutical Society of Northern Ireland (PSNI),9., 10. as part of a broader move in the early 2000s to make professional regulation in the UK more consistent, transparent, and focused on public protection. This came about as a direct result of a series of national scandals, many of which implicated the disciplinary functions of healthcare regulators.^11^(para. 1.82), ^12^ A 2007 government White Paper set the policy of reforming the regulation of health professional to make it fit for the 21st century.^13^ Among the reforms proposed were: separating professional bodies from regulatory functions; strengthening independence and public protection; increasing lay membership of regulatory councils; creating clearer accountability structures; and harmonising professional regulation across healthcare. Although the UK's system is far from perfect, having been accused in the past of being punitive and slow, it is nonetheless both proactive and reflexive in addressing its own shortcomings.^14^, ^15^, ^16^

## 2 Who regulates pharmacists?

Pharmacy is unique among regulated healthcare professions in the United Kingdom of Great Britain and Northern Ireland (UK), in that there are two statutory regulators. Pharmacists in Great Britain (GB) register with the General Pharmaceutical Council (GPhC), while those in Northern Ireland (NI) must be members of the PSNI.^9^^(art. 4),^^17^^(art. 2)^

The reasons for this are largely historical. Long-established healthcare regulators were created by Acts of parliament that applied to the UK, while newer professions were deliberately established at the UK-level for reasons of consistency. The General Medical Council (GMC), for example, was initially created (as the General Council of Medical Education and Registration of the United Kingdom) in 1858,^18^^(s. III)^ and the Health and Care Professions Council (HCPC) was established in 2001 under provisions in the Health Act 1999.^19^^(s. 60)^ As the regulation of pharmacy across Ireland before partition in 1921 was undertaken by the Pharmaceutical Society of Ireland (PSI), Northern Ireland gained its own regulator under the Pharmacy and Poisons Order (Northern Ireland) 1925.^20^ Due, in large part, to the overlap of pharmacy law with medicines law and local health service delivery, and the complex legislation in multiple governments to effect the required structural changes, the PSI was not subsumed by the GPhC when it was created in 2010. As the GPhC is – by a factor of 30 – the larger of the two organizations,^a^ we will use it as a proxy for both for the remainder of this treatise.

The governing Council of the GPhC is appointed through a statutory public appointments process,^21^ with appointments formally made by the Privy Council, which is constituted of current or former members of government and senior judiciary. The involvement of the Privy Council ensures that regulatory powers, including professional discipline and public protection are exercised with democratic oversight independent of the profession being regulated. The GPhC's Council consists of 14 members, seven of which must be pharmacists or pharmacy technicians, and seven who must be lay members, who have never been registered health professionals.^22^^(art. 2)^

In the United States, the regulation of the pharmacy profession is undertaken at the state level, with each state responsible for the licensing of pharmacists within its borders. This responsibility is entrusted to State Boards of Pharmacy (SBPs). Members of SBPs are generally appointed through state government processes, with the exact method varying slightly by state. In most states, board members are nominated exclusively by the State Governor and confirmed by the state Senate or legislature. Some states, such as California, may additionally have a small number of appointments made directly by the State Legislature. Nearly all states are required to have a majority of pharmacists among their members. Several states, including Mississippi, have boards comprised entirely of pharmacists.^23^

## 3 Who disciplines pharmacists?

A significant difference between the systems is the way in which disciplinary authority is operationalised. The UK model clearly demarcates its regulatory and disciplinary functions across distinct entities. Disciplinary cases are brought by the Council to be adjudicated upon by the independently constituted Fitness to Practise Committee, with statutory safeguards to reinforce its separation from the Council. Members of the GPhC's governing council (the equivalent of the Membership of a US SBP) are explicitly prohibited from sitting on the Fitness to Practise Committee for the duration of their tenure and for a further four years afterwards.^24^^(rule. 7)^

In Britain, disciplinary hearings are determined by three-member panels drawn from a pool of approximately 40 Fitness to Practice Committee members. These individuals are appointed through a merit-based, competitive recruitment process rather than by political patronage. Selection criteria include strong intellectual and analytical capability, sound decision-making and judgment, a demonstrated commitment to fairness, equality, and diversity, and proven integrity, alongside an undertaking to adhere to the Principles of Public Life established by the Committee on Standards in Public Life.^25^ Panel members are remunerated for their part-time service, contributing to the competitive nature of appointments. Newly appointed members undertake several days of intensive training, focusing on the statutory and procedural frameworks governing hearings and the core competencies required for adjudicating disciplinary matters. Members are also expected to maintain and update their expertise through ongoing professional development, including guidance updates, e-learning, webinars, and other training resources, as well as participation in an annual training event.^24^^(rule 10)^

Competency-based appointments to statutory committees are mandated for in legislation, supported by established best practice and overseen by a defined governance process. The statutory framework also sets out safeguards against bias, prohibiting members from participating in cases where an actual or potential conflict of interest arises, and requires the disclosure and maintenance of a public register of members' private interests.^24^^(rule 19)^

By contrast, the US disciplinary framework sees a profession-dominant Board retain control over both governance *and* disciplinary functions. SBPs perform both membership and regulatory functions, creating potential conflicts of interests that can undermine independence and public confidence in pharmacy regulation. We have already outlined how those administering discipline SBPs are recruited as they are the same board members who direct the Board's general regulatory and operational functions.

In some States, adjudicative expertise may be deferred to Administrative Law Judges (ALJs),^26^ who may or may not be professional regulatory law specialists, final disciplinary determinations must still be approved by the SBP. To compound matters, pharmacy board members, who are given an additional layer of discretion in considering ALJs' recommended decisions are not subject to any statutory competency requirements in relation to professional disciplinary decision-making, nor to training.

## 4 How are they guided?

When addressing disciplinary matters, State Boards have minimal statutory guidance or clearly defined rules to follow.^2^ In practice, they must determine whether a professional has breached broadly worded practice legislation and impose discipline in the public interest. Although SBPs occasionally issue policy statements on disciplinary matters, these are often as ambiguous as the statutes they interpret and offer limited direction. Where boards do provide detail, it typically focuses on identifying conduct that constitutes misconduct rather than specifying the appropriate sanctions for practitioners found to have engaged in such behaviour.

In stark contrast to this, disciplinary hearings held under the GPhC are required to follow the Outcomes Guidance for its Fitness to Practise Committee,^27^ and to consider cases with reference to its Standards for Pharmacy Professionals.^28^ This guidance is extensive, detailed, and publicly available. A failure to apply it correctly, or a departure from it without clear justification, may result in a decision being overturned on appeal. The Outcomes Guidance identifies mitigating and aggravating factors that are directed toward a registrant's fitness to practise, rather than personal character or moral blameworthiness. The guidance also establishes indicative sanctions for categories of misconduct: for example, removal from the Register for sexual misconduct. This framework is supplemented by a substantial body of additional GPhC guidance, which provides the Committee with direction on matters such as the interpretation of statutory provisions, the conduct and responsibilities of committee members, the use of expert evidence, applications for voluntary removal,^29^ and the factors relevant to each available sanction.^30^, 31. All of this guidance is public, transparent, and readily accessible on the GPhC's website. For example, in its Outcomes Guidance, the GPhC sets out a clear and structured approach to cases involving sexual misconduct, promoting consistency of purpose and outcome.^27^^(paras. 6.2–6.7)^ In determining sanction, decision makers are steered away from lower-level responses such as warnings or conditions and towards suspension or removal from the register. Sexual misconduct and sexual offences are expressly identified as aggravating factors that may justify the most serious outcomes, including removal from the GPhC register.

## 5 Who watches the watchmen?

Healthcare regulators in the UK are overseen by the Professional Standards Authority for Health and Social Care (PSA). The PSA derives its oversight authority from the National Health Service Reform and Health Care Professions Act 2002.^32^^(s. 25)^ It has a mandate “to strengthen and co-ordinate the system of professional self-regulation” to protect the public. The PSA ensures consistency in how disciplinary functions of regulation are effected, not only between the UK's two pharmacy regulators, but also across all regulated healthcare professions.

The PSA is given power to review and appeal disciplinary decisions made by the GPhC and PSNI (and their equivalents for each healthcare profession). This power is derived from s. 29 of the National Health Service Reform and Health Care Professions Act 2002, which provides that:

“… the Authority may refer the case to the relevant court if it considers that *the decision is not sufficient* … *for the protection of the public* (emphasis added).”^32^

The outcomes of such appeals affect *all* of the UK's more-than-thirty regulated healthcare professions. For example, a case involving a registered nurse which reinforced that impairment must be assessed not only by whether the registrant poses a current risk to patients, but also in the wider public interest, is frequently cited in disciplinary cases involving doctors, dentists and pharmacists.^33^

There is no such interprofessional oversight in the US. Indeed, even within the single profession of pharmacy, there are 50 largely unconnected and uncommunicative State Boards. This potentially exposes the system to regulatory failures at both a state and national level. The National Association of Boards of Pharmacy (NABP) is the nearest equivalent to the PSA in the United States. The NABP is not a regulatory body and does not have oversight powers like those of the PSA: rather, it occupies an advisory role to state pharmacy boards. From the perspective of pharmacy discipline, it maintains a national database of disciplinary and administrative information reported by the member boards of pharmacy on actions taken against pharmacists. This information, however, is only accessible to boards of pharmacy, and its purpose is to “determine the acceptability and qualifications of pharmacists who request the transfer of examination scores and licenses into other states or jurisdictions”, not to create an interstate disciplinary record.^34^ Although it offers pharmacist licensure transfer and verification services, these are entirely reliant upon voluntary reporting by each board of pharmacy. Further, investigations found that hospitals regularly under-report disciplinary issues with healthcare professionals, and that a considerable number of practitioners had agree to concessions so that no reportable action would be taken with their State Board.^35^ Research on physician discipline has already identified a similar lack of consistency between US States across the regulatory landscape for medical doctors.^7^

## 6 The disciplinary process from allegation to determination

To compare the procedural aspects of pharmacy discipline in the US and UK, respectively, we will juxtapose a single state which is broadly representative of the US model and comparable in key respects to those of the General Pharmaceutical Council, which is by far the larger of the UK's two pharmacy regulators. When necessary, reference will be made to other US states. California has been selected as the jurisdictional focus, given its detailed legislative framework, transparent disciplinary process and the scale and scope of its regulatory remit. The California State Board of Pharmacy (CSBP) operates under a clear statutory mandate to ensure public protection as its “highest priority … in exercising its licensing, regulatory, and disciplinary function.”^36^^(s. 4001(1))^ Enforcement activities are highlighted as essential for the board to meet its public protection mandate,^37^ and it has authority to impose sanctions on a licensee “whose case has been heard by the board and found guilty.^36^^(s. 4300(b))^

The disciplinary process can be categorised into two basic activities: investigation and adjudication.

### 6.1 Investigation

The investigation processes for pharmacist misconduct on each side of the Atlantic share several important similarities in their overarching structure and complaint handling procedures.^38^ Both jurisdictions begin their disciplinary procedures with the receipt of a complaint, usually from a member of the public or another professional body. In each system, these complaints are subject to an initial assessment to determine whether there is sufficient evidence to justify further investigation. Both systems incorporate an early screening stage designed to filter out unfounded or insufficient allegations before resources are committed to a full investigation. Such investigations are typically carried out by CSPB or GPhC employees who are qualified pharmacists.^37^^(p. 33),^^9^^(art. 8)^

In the event that an inspector determines that a violation has been committed at the conclusion of the inspection or investigation, the matter is reviewed and may be dealt with at that stage, usually with the issuance of a reprimand of the agreement of undertakings.^37^^(p. 33),^^39^^(rule 9)^ If, however, it cannot be disposed of as a minor violation, it must be escalated for further investigation either by California's Office of the Attorney General or the GPhC's Professional Regulation Team, and subsequent adjudication at an adversarial hearing. It is in the management of such hearings that the most marked differences – both between US States and between the US and UK – are most evident.

### 6.2 Adjudication

In California, a disciplinary determination is reached by one of two means: a settlement agreement; or an adversarial hearing. It is at this stage that the first obvious difference between the American and British process presents itself. The GPhC do not agree negotiated settlements with pharmacists referred for adjudication by its Fitness to Practise Committee. Conversely, US State Boards are actively encouraged to settle.^7^ Pharmacists wanting to avoid a public trial may favour negotiated orders, and SBP members – as unpaid volunteers – may also prefer to approve negotiated settlements than to take yet more time off from their professional lives to hear a case.

The format of a hearing in California may vary depending on the type of breach or misconduct being considered. In relation to criminal convictions, a hearing must be “before an administrative law judge, a committee of the [State Board of Pharmacy] sitting with an administrative law judge, or the board sitting with an administrative law judge … and [is] subject to review by the board, at its discretion.”^36^^(s. 4311(5))^ The state board itself determines whether the administrative law judge is to hear the case alone or whether the board itself is to hear the case with a judge present.^40^^(s. 11512(a))^

This format does not apply across all states. In Texas, for example, contested disciplinary proceedings are referred to the State Office of Administrative Hearings^41^^(s. 2003.021),^^26^^(s. 281.30)^, which conducts hearings under Title 5 of the Labour Code,^41^^(s. 2003.021(c))^. An administrative law judge (ALJ) conducting a contested case hearing must consider applicable state board rules and policies in conducting the hearing, but state board may not supervise the administrative law judge.^41^^(s. 2001.058)^

In New York, contested cases are heard without any judicial input. Rather, they are considered by a panel consisting of at least two pharmacist members of the New York State Board of Pharmacy, and at least one who is either a lay member of the board, or a member of the state board for another licensed profession.^42^^(6510(3))^ At the conclusion of the hearing, the panel submits a written report for review by the Professional Practice Committee of the state's Education Department, at which hearing the licensee may appear, with legal representation, at the Committee's discretion. This Committee in turn, prepares a report for the Board of Regents, who considers it in conjunction with the report of the hearing panel and the hearing transcript, to “decide whether the licensee is guilty or not guilty on each charge … what penalties, if any, to impose … [and] issue an order to carry out its decisions.”^42^^(s. 6510(4)(c))^

There is little consistency across SBP regulations, and where it does exist it is usually because one State's Legislative Counsel have used the statue of one state as a template for their own.^43^

The GPhC's Fitness to Practise Committee, in contrast, is a model of consistency. As with all GPhC's statutory committees, it must be quorate with at least three members comprising of a legally qualified chair or deputy chair, a lay member and a registrant member.^24^^(rule 18(1))^ This has the effect of ensuring that two members of the panel of three are not registered pharmacists.^38^

It makes its determinations in a 3-stage sequence: finding firstly on the facts of the case; before determining if the registrants fitness to practice is impaired based on the facts found, and – if so – what sanction, if any, should be imposed.^39^^(rule 31(10–14))^ All charges must be assessed in terms of whether the pharmacist's fitness to practise is (currently) impaired. It is unlikely that a pharmacist would appear before the Committee were there not a strong chance that they had committed an act of misconduct based on the facts to be presented. More important is whether such a finding impairs their fitness to practice going forward. A transgression in the past that is easily remediable – for example, a gap in the pharmacist's knowledge base – and which *has* been remedied in the meantime does not necessarily impair a pharmacist's fitness to practice in the present.^44^ Conversely, there are forms of misconduct that are “fundamentally incompatible with being a registered professional.”^27^^(para. 4.3)^ Fitness to practise is a key concept, which refers to whether a professional has the skills, knowledge, health, behaviour, and character required to practise safely and effectively without posing a risk to patients or public trust. Deliberating misconduct in these terms prevents emotional decision-making, by extricating the penalty from the offence, and reframing it in terms of patient safety and public trust.

Sanctions are considered sequentially, beginning with the least restrictive outcome, to decide whether each adequately address public protection and public interest considerations, guided by published Outcomes Guidance.^27^ In reaching a decision on what outcome to impose, the committee is directed to “give appropriate weight to the wider public interest” which should outweigh “the consequences for the professional”.^27^^(para 5.7)^

## 7 Discussion

The remit of pharmacy discipline in the US and UK is essentially identical: both are tasked with protection of the public and the maintenance professional standards. However, there are clear differences in approaches to achieving those aims, which raise important questions about rigour, independence and consistency in disciplinary proceedings, and whether there is a correlation between procedure and disciplinary outcomes.

### 7.1 Public interest & regulatory capture

The American system of discipline potentially undermines the foundational pillars of regulation, particularly that of public interest and public protection, potentially posing serious risk of “regulatory capture”,^45^^(p. 13)^ in which regulation disproportionately serves the interest of the profession it regulates and not the public, which it is mandated to protect. The NABP lends support to this proposition, observing that: “The practice of pharmacy, from time to time, has been erroneously viewed, even by government agencies, as a commercial business rather than a profession.”^46^^(s. 102)^ In light of this, it has reasserted public interest as the overriding objective of pharmacy regulation. The NABP attempts to provide some consistency in pharmacy regulation between states: however, it is a voluntary, non-profit organisation and not a regulatory body; it does not report to, nor derive authority from Congress. There is currently no independent or federal-level agency that oversees or coordinates the regulatory functions of the discrete state boards of pharmacy.

Our understanding of the concept of public interest remains stuck by historical assumptions embedded in its origins. In exploring the historical context of healthcare regulation, Adams found that “[i]t was deemed to be in the public's interest to grant authority and privileges to educated white men who could take on positions of social and community leadership – as long as they were properly educated and trustworthy.”^47^ A shift occurred with the introduction of “public members and state actors to regulatory boards, to increase public involvement and decrease abuses of professional power.”^47^^(p. 5)^ Further shifts saw movements to “encompass cost containment and efficiency”, and more recent advances see the concept of public interest as being “defined in terms of efficiency, accountability, governance transparency and tight control of professional practice.”^47^^(p. 6)^ In the UK, the prevailing rhetoric is that public protection is about “protecting the public *from* professionals” (emphasis in original).^47^^(p. 6)^ In the United States, however, there is evidence to suggest that remnants of older concepts of the public interest remain.

There is growing concern that the fragmentary nature of professional regulation in the US, albeit “arguably beneficial historically [in] providing regulation responsive to local needs and conditions”, is [now] detrimental to “regulatory bodies' transparency, accountability and … [robust] disciplinary proceedings”,^47^^(p. 5)^ and that “[t]he American people deserve a regulatory system that works for them, not against them.”^48^

Regulatory capture can be defined for our purposes as “the result or process by which regulation, in law or application, is consistently or repeatedly directed away from the public interest and toward the interests of the regulated industry, by the intent and action of the industry itself.”^45^^(p. 13)^ In exploring the necessity of intent in diagnosing valid regulatory capture and how it might manifest in practice, Carpenter explains that it includes “an attempt to stack the deck of an institutional process, or … an attempt to influence frames, assumptions, and worldviews of regulators or professionals involved in regulation.”^45^^(p. 62)^ When looking at board composition, for example, the deck is certainly stacked in favour of pharmacists, who, on average, outnumber public (lay) members by a ratio of 3:1.^49^^(p. 6)^ This issue is further complicated by the fact that most SBP members are gubernatorial appointments.^49^^(p. 7)^

### 7.2 Bounded accountability

The issue of regulatory capture is one that warrants investigation in the sphere of pharmacy discipline, but it is not the only one raised by the current structure of US regulation. Recent research on transparency and accountability in professional discipline has led to the development of the concept of “bounded accountability”.^50^ Bounded accountability refers to regulators imposing only limited discipline, due to constraints placed on the exercise of accountability. Just as regulatory capture might explain the dominance of pharmacists on state pharmacy boards, so might bounded accountability illustrate why those pharmacists feel able to administer disproportionately mild sanctions to those they are charged with holding to account. Constraints on Board accountability may include: “information asymmetries, shared professional beliefs, bureaucratic inefficiencies, and interpersonal emotions. It has been noted that “professionals and their disciplinary bodies are often ineffective at sanctioning misconduct among their ranks”, and heightened transparency measures, with the inclusion of public members in professional bodies, has had little to no impact in combating bounded accountability.^50^ Scholarship informing this research found that “a system biased in favour of [the profession it regulates] will produce too-lenient outcomes.”^6^ This highlights the need for empirical research into the impact of “heightened transparency measures” on “how and when professional bodies hold their members accountable.”^50^

Comparative studies on the regulation of doctors found that the US system of self-regulation is excessive by dint of, among other things, the composition of state medical boards, weighted in favour of the profession, and the absence of any meaningful demarcation between licensing and disciplinary functions.^6^, ^7^ This system was found to produce fewer severe sanctions for doctors involved in misconduct proceedings than their contemporaries in the UK. With respect to doctors, it has been asserted that “the American licensing system disciplines professionals too infrequently, too late, and too leniently to adequately protect the public.”^7^ The issue of bounded accountability remains a live and valid concern in professional regulation; therefore, our proposed program of research aims to extend the inquiry, and test whether similar patterns exist in pharmacy regulation.

### 7.3 The Model State Pharmacy Act: unrealized potential?

Many State Pharmacy Boards' public protection function “is hampered by limitations in their legal authorities, *administrative processes*, and resources [emphasis added]”.^51^ This legitimises the concern that outcomes may be affected as much as by process as by the underlying conduct itself. California's processes – both for populating the CSBP and executing its disciplinary functions – have significant implications for adequately realising the regulatory mandate. The GPhC's clear separation of its adjudicatory and regulatory functions, coupled with an emphasis on lay participation, supports the principles of public protection and confidence in the system. California's model, though it secures some semblance of independence with the involvement of ALJs, is driven by the profession and invites regulatory capture. It remains an open empirical question as to whether these differences produce different outcomes and/or different perceptions of fairness among the public and registrants.

While it attempts to introduce a degree of consistency in the drafting of pharmacy legislation, the NABP's Model Act falls short of addressing the structural inconsistencies between State Boards. In its section on board membership, for example, the Model Act provides that:

The board of pharmacy shall consist of ___________ members, __________ of whom shall be a representative of the public, ___________ of whom shall be a certified pharmacy technician, and the remainder of whom shall be pharmacists … Individual states may wish to consider a board composition that represents the diversity of the population and the profession within the state.^46^^(s. 203)^

This does not address any imbalance in representation on boards by – for example – explicitly stating that members of the public must not be outnumbered by professional appointees, as is specified in British legislation.^24^^(rule 3)^ Rather it yields to individual State Governors to “determine what attributes an individual should possess in order to meaningfully serve on a Board of Pharmacy” reasoning that since nominations are recommendations only, the Governor retains complete discretion regarding the appointee.^46^^(s. 203)^ Indeed, the NABP's 12-member Executive Committee is elected entirely from gubernatorial States Board appointees, consisting almost entirely of pharmacy professionals (10 pharmacists and 1 certified pharmacy technician), with a single member from a legal background.

The potential for regulatory capture on SPBs is, again, ignored in the Model Act, where it is stated that “[a]ny investigation, inquiry, or hearing [of the state board] may be held or undertaken by or before any member or members of the board and the finding … shall be deemed to be the order of said board.”^46^^(s. 213(2)(d))^ The Act is silent as to quoracy for legitimacy and efficacy: there is no recommendation that disciplinary panels should be properly constituted with balanced representation, including public members. This leaves the gates wide open for the very issues this research seeks to examine. The Model Act provides a good foundation on which to build a usable template for pharmacy legislation: however, it is loosely drafted, and it is apparent from earlier scholarship that SBPs differ in which of its provisions they opt to incorporate into their legislation and how they implement these.^52^

Medical regulation in the UK was fragmented before the General Medical Council (GMC) was established. This was “a legislative achievement … following decades of negotiations and effort, and resulting from collaboration among several medical organizations and government leaders, with the goal of establishing some uniformity in medical regulation.”^47^ A similar collaborative effort, starting with the careful drafting of a meaningful Model Act, is crucial to unite the fragmented domain of pharmacy regulation in the US, and to contribute to scholarship on regulatory reform.

## 8 Research agenda

Before any meaningful attempt to reform pharmacy discipline in the US can be attempted, it is necessary to qualify and quantify current concerns, and to examine whether, and how, the prevailing governance structure of the disciplinary process influence the severity and consistency of penalties imposed on pharmacists. We therefore propose a mixed-methods approach.

In the first instance, it is necessary to compare the discrete legislative frameworks for pharmacy regulation across all 50 US States. This should include legal statutes, relevant case law and any rules or guidance provided for decision-makers. Given that the UK model is proposed as an exemplar, the coding frame should be based on the current UK statue book. Laws on the composition of pharmacy boards (including appointing authority, termination, qualifications and ratio of professional to lay representation), definitions of misconduct or unprofessional conduct and sanctions available to boards must be collated, coded and compared.

To determine if differences in regulatory structure between US States, and between the US and UK are associated with different outcomes, a statistical analysis of penalties issued must first be undertaken. The National Practitioner Data Bank (NPDB) is a web-based repository of reports containing information on each SBP's Disciplinary Action Reports (DARs) for its pharmacists. It was established by Congress in 1986 as a tool to prevent healthcare practitioners from moving from state to state without disclosure of previous malpractice. The NPDB Public Use Data (PUD) file is updated four times a year. The current version contains disclosable reports received from 1 September 1990 through 31 December 2025. Fitness to practice determinations made by the GPhC and PSNI are published on their respective websites for twelve months from the date of decision. Historical data from the UK can been obtained by a request made under s. 8 of the Freedom of Information Act.

Where significant differences are identified, an ex-post impact approach should be applied toward the determination if any legal idiosyncrasies, either independently or in combination, have a measurable effect on outcomes. The questions of whether a SBP dominated by pharmacist members leads to regulatory capture and bounded accountability, or if the separation of a board's governance and disciplinary functions produces outcomes more aligned with a public protection remit, should be interrogated at this stage. A codebook approach to the thematic analysis of similar cases from jurisdictions with differing legal foundations at this stage would allow for the development of an understanding of how rules and procedures affect outcomes in support of any statistically significant differences found.^53^ Transcripts or recordings of most cases can be obtained for a nominal fee under each states' freedom of information laws.

For a more nuanced understanding of how the disciplinary process is experienced in both the US and UK, the research should also include a reflexive thematic analysis of semi-structured interviews informed by the above with key stakeholders,^54^, ^55^ including: SBP members; GPhC and PSNI panelists; pharmacist who have been subject to disciplinary proceedings; and legal counsel from both sides of the adjudication. Diverse perspectives on the topic would help provide some insight into how the process operates in practice.

The accumulated knowledge as to what legislative provisions and guidelines drive discipline in service of patient safety and public trust could then be applied in drafting a less vague, more universal Model Act, which could be applied more uniformly across the 50 states, or – indeed – be adopted wholesale by a theoretical “51st state”.

## CRediT authorship contribution statement

**Denzil Johnson:** Writing – original draft, Investigation, Formal analysis, Data curation. **Nkiruka Umaru:** Writing – review & editing, Supervision, Methodology. **Cathal T. Gallagher:** Writing – review & editing, Writing – original draft, Supervision, Resources, Project administration, Methodology, Investigation, Formal analysis, Conceptualization.

## Declaration of competing interest

The authors declares that they have no competing interests.

## Footnotes

a As of 31 December 2024, there were approx. 93,000 pharmacy professionals registered with the GPhC, compared to 3100 with the PSNI.
